# The Metric System

**Published:** 1878-09

**Authors:** 


Inasmuch as the international decimal or metric system of weights and measures will be adopted by most, if not all, civilized nations in a short time, we think proper to call the attention of our readers to the fact frequently. The centigrade thermometer is also proposed instead of Fahrenheit, which is used exclusively in this country. We give below tables of metrical weights and measures, and their equivalents in our present system, with scales for the centigrade thermometer and the measure of length in meters:

CENTIMETERS AND MILLIMETERS.

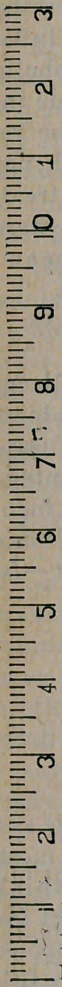

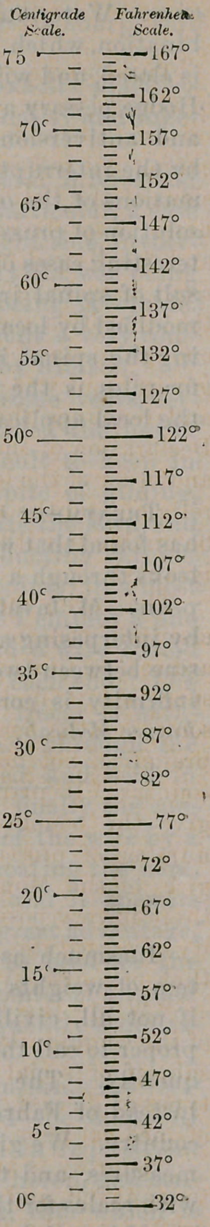

### FEDERAL MONEY.

10 mills make one cent.

10 cents make one dime.

10 dimes make one DOLLAR.

10 dollars make one eagle.

### WEIGHTS.

| | | | |
|---|---|---|---|
| 10 milligrams | 1 centigram | =gr. | 0.15. |
| 10 centigrams | 1 decigram | =gr. | 1.50. |
| 10 decigrams | 1 GRAM | =gr. | 15.43. |
| 10 grams | 1 dekagram | =ʒ | 2 57. |
| 10 dekagrams | 1 hectogram | =℥ | 3.21. |
| 10 hectograms | 1 kilogram | =℔. | 2.67. |
| 10 kilograms | 1 myriameter | =℔. | 26.70. |

### CAPACITY.

| | | | |
|---|---|---|---|
| 10 milliliters | 1 centiliter | =flʒ | 2.56. |
| 10 centiliters | 1 deciliter | =fl℥ | 3.20. |
| 10 deciliters | 1 LITER | =o. | 2.00. |
| 10 liters | 1 dekaliter | =c. | 2.50. |
| 10 dekaliters | 1 hectoliter | = " | 25.00. |
| 10 hectoliters | 1 kiloliter | = " | 250.00. |

### LENGTH.

| | | | |
|---|---|---|---|
| 10 millimeters | 1 centimeter | =inch | 0.39 |
| 10 centimeters | 1 decimeter | = " | 3.90 |
| 10 decimeters | 1 METER | =ft. | 3.25 |
| 10 meters | 1 dekameter | =yds. | 10.83 |
| 10 dekameters | 1 hectomerer | = " | 108.33 |
| 10 hectometers | 1 kilometer | = " | 1083.33 |
| 10 kilometers | 1 myriameter | =miles | 6.15 |

